# Evaluation of a curcumin analog as an anti-cancer agent inducing ER stress-mediated apoptosis in non-small cell lung cancer cells

**DOI:** 10.1186/1471-2407-13-494

**Published:** 2013-10-24

**Authors:** Zhiguo Liu, Yusheng Sun, Luqing Ren, Yi Huang, Yuepiao Cai, Qiaoyou Weng, Xueqian Shen, Xiaokun Li, Guang Liang, Yi Wang

**Affiliations:** 1 Chemical Biology Research Center, School of Pharmaceutical Sciences, Wenzhou Medical University, University Town, 325035 Wenzhou, Zhejiang, China; 2 Department of general surgery, the First Affiliated Hospital of Wenzhou Medical University, Wenzhou, Zhejiang, China

**Keywords:** Curcumin analogs, Anti-cancer, ER stress, Non-small cell lung cancer, CHOP

## Abstract

**Background:**

Recent advances have highlighted the importance of the endoplasmic reticulum (ER) in cell death processes. Pharmacological interventions that effectively enhance tumor cell death through activating ER stress have attracted a great deal of attention for anti-cancer therapy.

**Methods:**

A bio-evaluation on 113 curcumin analogs against four cancer cell lines was performed through MTT assay. Furthermore, real time cell assay and flow cytometer were used to evaluate the apoptotic induction of (1E,4E)-1,5-bis(5-bromo-2-ethoxyphenyl)penta-1,4-dien-3-one (B82). Western blot, RT-qPCR, and siRNA were then utilized to confirm whether B82-induced apoptosis is mediated through activating ER stress pathway. Finally, the *in vivo* anti-tumor effect of B82 was evaluated.

**Results:**

B82 exhibited strong anti-tumor activity in non-small cell lung cancer (NSCLC) H460 cells. Treatment with B82 significantly induced apoptosis in H460 cells *in vitro* and inhibited H460 tumor growth *in vivo*. Further studies demonstrated that the B82-induced apoptosis is mediated by activating ER stress both *in vitro* and *in vivo*.

**Conclusions:**

A new monocarbonyl analog of curcumin, B82, exhibited anti-tumor effects on H460 cells via an ER stress-mediated mechanism. B82 could be further explored as a potential anticancer agent for the treatment of NSCLC.

## Background

Recent advances have highlighted the importance of the endoplasmic reticulum (ER) in cell death processes. Perturbation of ER functions leads to ER stress, which has been previously associated with a broad variety of diseases, while prolonged ER stress can activate apoptotic pathways in damaged cells [1]. For this reason, pharmacological interventions that effectively enhance tumor cell death through activating ER stress have attracted a great deal of attention for anti-cancer therapy.

Curcumin is an active phenolic compound extracted from the rhizome of the plant *Curcuma longa*. Extensive research over the last half century has revealed various bio-functions of curcumin. Its anti-cancer effect has been seen in a few clinical trials, mainly as a native chemoprevention agent in colon and pancreatic cancer [2]. Recently, it was reported that curcumin exerts its pro-apoptotic effects by inducing ER stress in several tumor cells, including acute promyelocytic leukemia cells [3], human non-small cell lung cancer H460 cells [4], and human liposarcoma cells [5]. Although curcumin has an evident anti-cancer activity, rapid metabolism and low bioavailability have been highlighted as the major limitations in therapeutic applications [6]. To enhance metabolic stability and pharmacological potency, various curcumin analogs have been synthesized, among which, the mono-carbonyl analogs of curcumin (MACs) have been developed by our laboratory in the past six years. Without the central β-diketone moiety in curcumin structure, the MACs exhibit enhanced stability *in vitro* and an improved pharmacokinetic profile *in vivo*[7-9].

Advance in molecular biology has allowed a change in anti-cancer therapy trends, from classic cytotoxic strategies to the development of new therapies which target the special apoptosis response in tumor cells. The aim of our laboratory is to find anti-cancer therapeutic agents with relatively new mechanism. In continuation of our ongoing research, we evaluated here 113 synthetic MACs for their anti-proliferative effects, among which, the active compound (1E,4E)-1,5-bis(5-bromo-2-ethoxyphenyl)penta-1,4-dien-3-one (B82) was further examined as an excellent anti-tumor agent both *in vitro* and *in vivo*. Importantly, our results showed that B82 may induce cancer cell apoptosis via activating ER stress-mediated apoptotic pathway.

## Methods

### Cell lines and reagents

Human breast cancer cell line MCF-7, carcinomic human alveolar basal epithelial cell line A549, human lung carcinoma cell line H460, human liver carcinoma cell line HepG2, and normal human lung (bronchial) epithelial cell line BEAS-2B were purchased from ATCC (*Manassas, VA*); normal human liver cell line HL-7702 was purchased from Shanghai Institute of Life Sciences Cell Resource Center (*Shanghai, China*). The cells were cultured in RPMI 1640 medium (*Invitrogen, Carlsbad, CA*) supplemented with 5% heat-inactivated FBS (*Atlanta Biologicals Inc., Lawrenceville, GA*) and 100 U/mL penicillin and streptomycin (*Mediatech Inc., Manassas, VA*), and incubated at 37°C with 5% CO_2_. FITC Annexin V apoptosis Detection Kit I was purchased from BD Pharmingen (*Franklin Lakes, NJ*). Anti-CHOP, anti-GRP 78, anti-GAPDH, anti-Actin, anti-Bcl-2, anti-Cyclin D1, anti-COX-2, goat anti-rabbit IgG-HRP, mouse anti-goat IgG-HRP antibodies were from Santa Cruz Biotechnology (*Santa Cruz, CA*), and anti-cleavaged caspase-3 was from Cell Signaling Technology (*Danvers, MA*). Ambion RNAqueous kit was purchased from Applied Biosystems Inc. (*Foster City, CA*). Caspase 3 Activity Assay Kit was from Beyotime Biotech (*Nantong, China*).

### Chemistry

Curcumin was purchased from Sigma (*St. Louis, MO*). Curcumin analogues **1**–**113** were synthesized by our laboratory, and were reported in our previous articles with their anti-inflammatory activities [9-12]. The names and structures of these compounds were shown in Additional file 1: Table S1. Before used to the biological experiments, compounds were purified by re-crystallization or silica gel chromatography to reach the purity higher than 97.0%. In *in vitro* experiments, compounds were used in DMSO solution, when the final concentration of DMSO in cultural medium is 0.1%.

### Methyl thiazolyl tetrazolium (MTT) assay

All experiments were carried out 24 h after cells were seeded. Tested compounds were dissolved in DMSO and diluted with 1640 medium to final concentrations of 0.3, 1.25, 5, 20, and 80 μM. The tumor cells were incubated with test compounds for 72 h before the MTT assay. Curcumin was applied as the positive control.

### Dynamic monitoring of H460 cell proliferation using the RT-CES system

The real time cell electronic sensing assay is based on electrical impedance readings in cell monolayers plated in wells containing built-in gold electrodes. We have used the ACEA RT-CES analyzer, 8 well e-plates, and the integrated software from Acea Biosciences Inc. (San Diego, CA). Cells were plated at a density of 30,000 cells/well in 100 μl of medium. The analyzer and the installed plates were placed in a standard cell culture incubator, at 37°C in a humidified atmosphere of 5% CO_2_. Cells were allowed to adhere to plates overnight. After cell seeded, the analyzer was programmed to take readings during 0–96 h; and B82 at 2.5 or 10 μM was added to the medium at 40 h after incubation. Data were recorded and analyzed using the integrated software. The cell index is a quantitative measure of the spreading and/or proliferative status of the cells in an electrode-containing well.

### Cell apoptosis analysis

H460 cells were placed in 60-mm plates for 12 h, and then treated with varying doses (2.5, 5 and 10 μM) of compound B82, curcumin (10 μM) or vehicle (DMSO, 3 μL) for 12 h. Cells were then harvested and stained with Annexin V and propidium iodide (PI) in the presence of 100 mg/mL RNAse and 0.1% Triton X-100 for 30 min at 37°C. Flow-cytometric analysis was performed using FACScalibor (*BD, CA*).

### Western blot analysis

Cells or homogenated tumor tissues were lysated. The protein concentrations in all samples were determined by using the Bradford protein assay kit (*Bio-Rad, Hercules, CA*). Lysates were then analyzed through western blot assay, and the immunoreactive bands were visualized by using ECL kit (*Bio-Rad, Hercules, CA*).

### RNA isolation and real-time quantitative PCR

Total mRNA was isolated from the treated cells using Ambion RNAqueous kit after treatment with compounds or control DMSO. The High-Capacity cDNA Archive Kit was used to obtain first-strand cDNAs of mRNAs. The mRNA levels of CHOP, XBP-1, ATF-4 and GRP78 were quantified by specific gene expression assay kits and primers on iQ5 Multicolor real-time PCR detection system (*Bio-Rad, Hercules, CA*) and normalized to internal control β-actin mRNA.

### Caspase-3 activation assay

Caspase-3 activity was determined using a Caspase-3 activity kit (*Beyotime institute of biotechonoly, Nantong*, *China*) according to the manufacturer’s protocol. The OD value representing caspase-3 activity was detected with a microplate spectrophotometer (*MD, Sunnyvale, CA*) at 405 nm. The caspase-3 activity was normalized by the protein concentration of the corresponding cell lysate, and was expressed in enzymatic units per mg of protein.

### Construction of lentiviral siRNA for CHOP

The sense sequence of the siRNA cassettes specifically targeting the nucleotides of CHOP was designed through siRNA Target Finder (*Ambion, Austin, TX*). A two-step polymerase chain reaction (PCR) strategy was performed using two separate reverse primers to generate a siRNA expression cassette (SEC) consisting of human U6 promoter and a hairpin siRNA cassette plus terminator and subcloned into pGL3.7 vector, which encodes the CMV-promoted EGFP (enhanced green fluorescent protein) marker as internal control. The resulting lentiviral siRNA vector was confirmed by restriction enzyme digestion and DNA sequencing. The sequence of CHOP siRNA is 5′-GCAGGAAATCGAGCGCCTGAC-3′. The recombinant lentiviruses were produced by transient transfection of H460 cells using FuGene 6 Transfection reagent (*Roche Inc., Nutley, NJ*). Titers were determined by infecting H460 cells with serial dilutions of concentrated lentivirus and counting EGFP-expressing cells after 48 h under fluorescent microscopy.

### *In vivo* antitumor study

All animal experiments complied with the Wenzhou Medical College Policy on the Care and Use of Laboratory Animals (Wenzhou Medical University Animal Policy and Welfare Committee, 201100009). Five-week-old to six-week-old athymic nu/nu BALB/cA male mice (18–22 g) were purchased from Vital River Laboratories (*Beijing, China*). Animals were housed at a constant room temperature with a 12:12 hr light/dark cyclic, and fed a standard rodent diet and water. H460 cells were harvested, and mixed with Matri Gel in 1:1, and then injected subcutaneously into the right flank (2 × 10^6^ cells in 200 μL PBS) of 7-week-old male BALB/cA nude mice. One day after injected with H460 cells, treated mice were intraperitoneally (i.p.) injected with a water-soluble preparation of B82 in PBS at dosage of 5 mg/kg/day for 28 days, whereas control mice were injected with liposome vehicle in PBS. The tumor volumes were determined by measuring length (l) and width (w) and calculating volume (V = 0.52 × l × w^2^) at the indicated time points. The tumor weights were recorded on the day of scarification.

### Immunohistochemistry

The harvested tumor tissues were fixed in 10% formalin at room temperature, processed and embedded in paraffin. Parraffin-embedded tissues were sectioned (5 μm thick). Tissue sections were primarily stained with indicated antibodies. The signal was detected by biotinylated secondary antibodies, and developed in DAB. Quantity assay of the immunochemistry data was obtained with Image-Pro Plus 6.0 (*Media Cybernetics, Inc., Bethesda, MD*).

### Statistical analysis

All experiments were assayed in triplicate (n = 3). Data are expressed as means ± SEM. All statistical analyses were performed using GraphPad Pro. Prism 5.0 (*GraphPad, San Diego, CA*). Student’s t-test was employed to analyze the differences between sets of data. A *p* value < 0.05 was considered significant.

## Results and discussion

### Anti-tumor evaluation of 113 curcumin analogs led to the discovery of active compound B82

**Figure 1:**
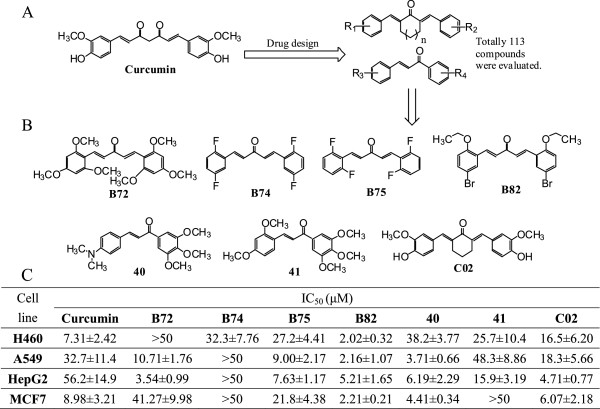
**Structures and cytotoxicity of synthetic curcumin analogs. A**. Drug design of 113 mono-carbonyl analogs of curcumin from the lead curcumin; **B**. Chemical structures of seven active compounds; **C**. The IC_50_ values of listed compounds against four cancer cell lines.

It has been reported that curcumin possesses a wide-spectrum of anti-tumor properties. Our laboratory has been engaged in finding promising anti-inflammatory and anti-cancer agents from curcumin analogs. Due to the important role of β-diketone in the metabolic defect of curcumin, we designed a series of stable MACs by deleting β-diketone moiety. To date, more than 400 MACs have been synthesized. To find the potential anti-cancer candidates from curcumin analogs, we tested the cytotoxicity of 113 MACs (shown in Figure 1A and Additional file 1: Table S1) in several tumor cell lines by MTT assay. The chemical structures and growth-inhibitory IC_50_ values of the best seven active compounds against H460, A549, HepG2, and MCF-7 cells were shown in Figure 1B and 1C. Among these compounds, B82 exhibited the strongest anti-tumor activity against H460 (2.02 ± 0.32 μM), A549 (2.16 ± 1.07 μM), and MCF-7 cells (2.21 ± 0.21 μM) compared with other tested compounds. B82 also exhibited a strong cytotoxicity in HepG2 cells (IC_50_ = 5.21 ± 1.65 μM).

The cytotoxic evaluation of these 113 compounds supported our previously published structure-activity relationship (SAR) analysis of MACs [13]. Most obviously, curcumin analogs with the acetone or cyclohexanone linker in the structures of MACs are beneficial to increasing the cytotoxic activity compared to the cyclopentanone linker. We also found that, in the chalcone-structure-containing MACs, the cytotoxic activity of compounds could be increased by the electron-donating substituents in both rings, especially at 4-position of ring A, and could be reduced through the induction of electron-withdrawing substituent, indicating that the electron-donating modification may be pharmacologically favorable for the anti-tumor drug design of this kind of MACs.

### B82 inhibited proliferation and induced apoptosis in H460 cells

**Figure 2:**
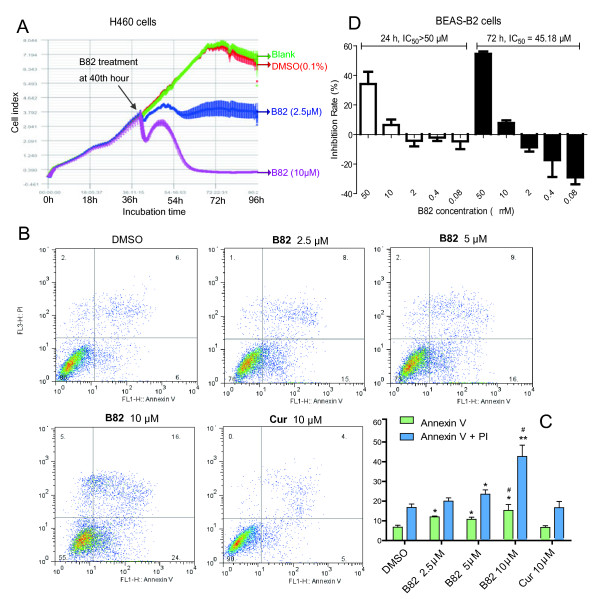
**The effects of B82 on H460 and BEAS-2B cells. A**. Inhibitory effects of B82 on the proliferation of H460 cells. H460 cells were seeded on laminin-coated plates of an ACEA RT-CES system at a density of 30,000/well and were continuously monitored up to 96 h. At the time point of 40 h, B82 at 2.5 or 10 μM and vehicle control DMSO were added into the corresponding wells (indicated by color and arrow). The data represent the mean values ± S.D. for triplicate wells. **B**. B82 induced cell apoptosis in H460 cells. H460 cells were treated with B82 or curcumin at indicated concentrations for 12 h, and then stained with Annexin V and PI, followed by detection using flow cytometry. The representative pictures are shown. **C**. The percentage of cells with early apoptosis and late apoptosis are shown (n = 4). Data are presented as the mean ± SEM. *p < 0.05, vs. vehicle control; # p < 0.05, vs. curcumin group. **D**. B82 affects the proliferation of BEAS-2B cells. Cells were treated with B82 at indicated concentrations for 24 h or 72 h, and the cell survival was determined using MTT assay, and the inhibitory rates and IC_50_ values were calculated.

Although our findings clearly show B82 to have anti-cancer properties, the mechanism involved is unknown. We extended our study to include cell proliferation in H460 cells. Inhibitory effects of B82 at 2.5 and 10 μM on the proliferation of H460 cells on laminin-coated plates were tested using the RT-CES system. As shown in Figure 2A, B82 treatment strongly suppressed the proliferation of H460 cells. The RT-CES assay is a convenient way to continuously determine cell number and cell activity and offers a full-range detection of B82-induced effects. Figure 2A showed a continuously change of H460 cells during 0 h-56 h after B82 treatment. At about 12 h after B82 addition, H460 cells were undergoing death or apoptosis. We next assessed the effect of B82 on the induction of apoptosis in H460 cells by flow cytometry. Figure 2B and 2C show that B82 dose-dependently increased H460 apoptosis after 12-h treatment. B82 at 10 μM induced a higher cell apoptosis rate (Annexin V^+^/PI^-^, 15.33 ± 2.96%) than that of curcumin (6.667 ± 0.88%). Human lung (bronchial) epithelial cell line, BEAS-2B, was used to determine whether B82 has effects on normal lung cells. Our data found that it showed much higher IC_50_ values toward BEAS-2B cells than H460 cells (Figure 2D), indicating possible anti-cancer selectivity and safety. In addition, B82 showed low cytotoxicity against human normal lung epithelial cell line MRC-5 (IC_50_ = 33.59 μM) and normal human liver cell line HL-7702 (IC_50_ = 37.86 μM).

### B82 activated ER stress-mediated apoptotic pathway

**Figure 3:**
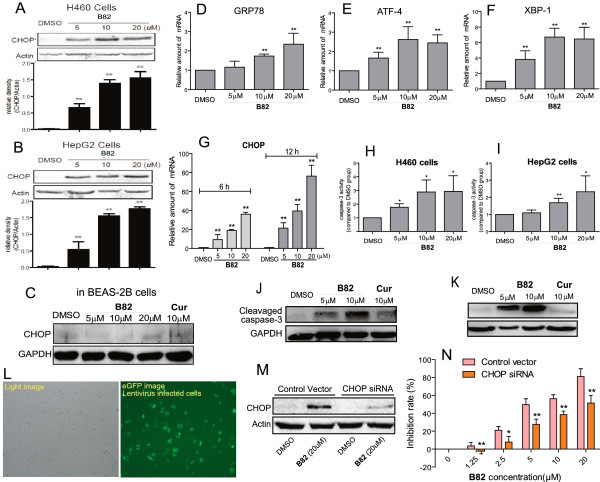
**B82 activates ER stress in H460 cells and CHOP knockdown inhibits B82-induced H460 cell apoptosis. A** and **B**. B82 induced CHOP expression in H460 **(A)** and HepG2 **(B)** cells. Cells were treated with B82 at the indicated concentrations for 12 h. Western blot results was calculated and represented as the percent of control (n = 3, **p < 0.01, vs. vehicle group). **C**. B82 could not induce CHOP expression in BEAS-2B cells. Cells were treated with B82 at the indicated concentrations for 12 h. The protein level of CHOP in total proteins was detected by western blot. **D-E**. B82 induced mRNA expression of GRP78 **(D)**, ATF-4 **(E)**, XBP-1**(F)**, and CHOP **(G)** in H460 cells. Gene mRNA expression were examined by RT-qPCR after 6- or 12-h treatment of B82. **H-K**. Cells were treated with B82 at indicated concentrations for 24 h. The caspase-3 activity in H460 **(H)** and HepG2 **(I)** cells was assayed. The total protein in H460 **(J)** and HepG2 **(K)** cells was extracted and cleavaged caspase-3 was examined by Western blot. **L-N**. H460 cells were transfected with CHOP siRNA virus. Forty-eight hours after transfection, the CHOP- and EGFP-expressing cells were counted using fluorescent microscopy **(L)**. Cells were treated with B82 (20 μM) for 12 h and then CHOP expression was determined by Western blotting **(M)**. Cells were treated with B82 at indicated concentrations for 48 h, and the cell survival was determined using MTT assay, and the inhibitory rates were calculated as percent of DMSO-treated cells **(N)**. * p < 0.05, ** p < 0.01, vs. vehicle control.

CHOP is considered as a marker of commitment of ER stress-mediated apoptosis [14]. We examined the effect of B82 on CHOP expression in H460 and HepG2 cells. As shown in Figure 3A-B, B82 is able to dose-dependently stimulate CHOP expression in both cell lines after 12-h treatment, indicating an evident activation of ER stress by B82 treatment. In comparison, B82 at the same concentrations could not induce the expression of CHOP in BEAS-2B cells (Figure 3C), suggesting a selectivity towards cancer cells of B82 activating ER stress. Glucose-regulate protein/immunoglobulin heavy chain binding protein (GRP78) is reported as the gatekeeper to the activation of the ER stress [15]. As shown in Figure 3D, treatment with B82 for 6 h significantly increased GRP78 mRNA level in H460 cells in a dose-dependent manner. Followed the gatekeeper, we tested the expressions of the downstream activating transcription factor 4 (ATF-4) and X-box binding protins-1 (XBP-1) in B82- or vehicle-treated H460 cells. RT-qPCR analysis revealed the significant increases in mRNA expression of both ATF-4 (Figure 3E) and XBP-1 (Figure 3F) in H460 cells after 6-h treatment with B82. We also confirmed the effect of B82 in induction of CHOP mRNA expression. Figure 3G gives the time course result showing that treatment with B82 for 12 h led to a more significant increase in CHOP mRNA expression compared to 6-h treatment.

Regarding to the structural feature in molecules inducing ER stress, B82 possesses two electron-donating substituents (ethoxyl group) in 2′-position of the benzene rings. These two ethoxy groups in B82 structure could increase the electrophilicity of the central α,β-unsaturated ketone as a Michael receptor. This supports the hypothesis that the cellular Michael adduct formation is responsible for ER stress activation via disrupting disulfide bond formation and consequently causing accumulation of unfolded proteins by the electrophilic olefine ketone [16]. These results are favorable for the anti-cancer drug design from curcumin analogs.

All upstream signals ultimately lead to caspase-3 activation to finish the execution of ER stress-induced apoptosis. The enzymatic activity of caspase-3 induced by B82 was assayed in both H460 (Figure 3H) and HepG2 (Figure 3I) cells. The activity of caspase-3 was increased in both cell lines after 24-h treatment with B82, in a dose-dependent way. Similar results were observed in the detection of cleavaged caspase-3. The cleavaged caspase-3 was significantly increased by the treatment with B82 for 24 h in both H460 (Figure 3J) and HepG2 (Figure 3K) cells, while curcumin at 10 μM could not activate caspase-3. In addition, other apoptotic markers, including cleaved-PARP (a downstream protein of caspase-3), P53, and Bax, were detected in B82-treated H460 cells by western blot method. As shown in Additional file 1: Figure S1, B82 at 10 μM could increase the expression of these three proteins after 24 h treatment. These data confirmed that B82 treatment induced apoptosis in H460 cells.

### Reduction of CHOP expression inhibits B82-induced H460 cell death

In order to further confirm that ER stress plays a critical role in the induction of H460 apoptosis by B82, we constructed the lentiviral siRNA for *CHOP* gene, which encoded the CMV-promoted EGFP marker as an internal control. As shown in Figure 3L, more than 75% of the H460 cells were transfected with lentiviral siRNA. Furthermore, the reduction of CHOP expression was confirmed by western blot assay in Figure 3M, showing that CHOP-siRNA significantly reduced B82-induced CHOP expression compared to the vector-transfected control. Finally, we treated CHOP siRNA-transfected H460 cells with B82 at indicated concentrations. Figure 3N shows that silencing CHOP expression in H460 cells significantly inhibited the cell apoptosis induced by B82, with a 4.14-fold increase in IC_50_ value from 4.63 μM to 19.17 μM (* P < 0.05, ** P < 0.01). These data demonstrate that B82-induced cell apoptosis is, at least partly, mediated by CHOP. However, other apoptotic mechanisms may also be involved in B82-induced apoptosis. The leading curcumin has been reported to exert anticancer effects by multitargeting mechanisms [2-5]. Despite that the siRNA CHOP could not completely wipe out ER stress activation, it only partly attenuated the apoptosis in H460 cells. Therefore, although this work only focuses on the ER stress-mediated apoptosis, further studies are necessary to establish such notions.

### B82 inhibited H460 tumor growth *in vivo*

**Figure 4:**
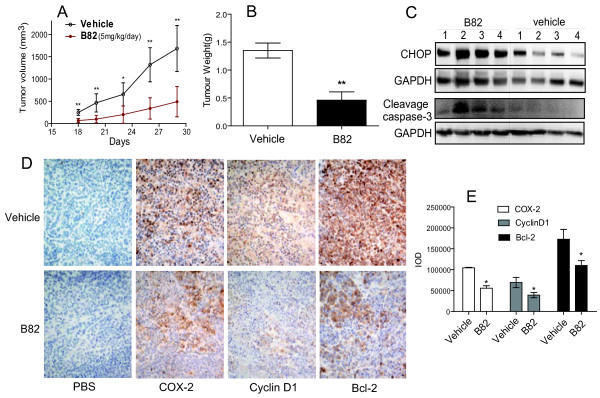
**B82 inhibited H460 xenograft tumor growth in athymic mice.** One day after injected with H460 cells, once daily i.p. injection of B82 (5 mg/kg) or vehicle was continued for the duration of the experiment. **A**. The volumes of the tumors (n = 10); **B**. The mice were sacrificed on day 29 and the tumors were weighed (n = 10). **C**. The tumor tissues from B82-treated or vehicle control mice were collected, proteins were extracted from tumors, and were subjected to western blot analysis for the determination of CHOP and cleavaged caspase-3 (n = 4). **D**. Tumor tissues (5 μm section) from each group (n = 4) were processed for immunochemistry analysis for Bcl-2, Cyclin D1, and COX-2. **E**. The column figure gives a quantitative analysis of relative amount of above proteins in tumor tissues (* p < 0.05, compared with control group).

We further investigated the *in vivo* anti-tumor effect of B82 using BALB/c nude mouse. A water-soluble preparation of B82 was prepared using a patented liposome technique as described in our previous paper [17]. Continuous once daily i.p. administration of mice with B82 at 5 mg/kg resulted in a significant inhibition of H460 xenografts tumor growth compared to that observed in vehicle group (Figure 4A). A reduction of tumor weight by B82 administration with treated versus control (T/C) of 65.9% was observed on day 28 after treatment (Figure 4B). B82 was well tolerated with no obvious weight loss over the treatment period, suggesting that it is relatively nontoxic to mice (data not shown).

To confirm the anti-tumor mechanism of B82 *in vivo*, we harvested the tumor tissues after the 28-day treatment. As shown in Figure 4C, CHOP expression was significantly increased in the B82-treated group compared to the vehicle control group. Similar results were observed in the expression of cleavaged caspase-3, indicating that the *in vivo* anti-tumor effect of B82 is also associated with ER-stress-mediated apoptosis.

In addition, we also detected other signaling markers possibly involved in the anti-tumor action of B82. The relative number of Bcl-2, cyclinD1, and COX-2 positive tumor cells was substantially less in tumors from mice treated with B82, when compared with control tumors (Figure 4D). Quantification of these stained samples showed 1.4- to 2-fold decreases in the number of oncogene-positive cells in the B82-treated groups compared with the control group (Figure 4E). Bcl-2 and COX-2 are identified as oncoproteins and play important roles in the mitochondria-mediated apoptotic pathway [18]. Cyclin D1 is a key protein in cell proliferation and is required for cell cycle G1/S transition. These results are also consistent with Figure 3N, in which silencing CHOP only partly attenuated the apoptosis in H460 cells. Thus, other apoptotic mechanisms may be also involved in B82-induced apoptosis that need be further investigated.

## Conclusions

In summary, a new monocarbonyl analog of curcumin, B82, was shown to exhibit anti-tumor effects on NSCLC via an ER stress-mediated mechanism. Although a series of curcumin analogs have been reported to exert anticancer effects both *in vitro* and *in vivo*, the molecular mechanism of these compounds are still unclear, and like curcumin, a majority of them showed multi-targeting mechanisms. The discovery of activation of ER stresss-mediated apoptosis by curcumin analog B82 may provide new strategy for curcumin-based anticancer drug design and development. In addition, we note that B82 also shows an excellent anti-inflammatory activity, and inhibits LPS-induced TNF-α and IL-6 release in mouse macrophages [10]. Further investigation should demonstrate the possible crosstalk and complementation between its anti-inflammation and anti-tumor properties. The new compound B82 could be further explored as a potential anticancer agent for the treatment of NSCLC.

## Abbreviations

ER: Endoplasmic reticulum; MCACs: Mono-carbonyl analogs of curcumin; UPR: Unfolded protein response; NSCLC: Non small cell lung cancer; CHOP: C/EBP-homologous protein/growth arrest and DNA damage-inducible gene 153; GRP78: Glucose-regulate protein 78; ATF-4: Activating transcription factor 4; XBP-1: X-box binding proteins-1.

## Competing interests

The authors declare that they no competing interest.

## Authors’ contributions

ZL carried out experiments, and performed data analysis. YS, LR, QW, XS carried out experiments. YH and YC contributed to supply testing compounds. XL participated in research design. GL participated in research design, performed data analysis, and drafted the manuscript. YW participated in research design, conducted experiments, performed data analysis, and drafted the manuscript. All authors read and approved the final manuscript.

## Pre-publication history

The pre-publication history for this paper can be accessed here:

http://www.biomedcentral.com/1471-2407/13/494/prepub

## Supplementary Material

Additional file 1: Table S1 Chemical information and sources of the tested 113 compounds. **Figure S1.** B82 treatment increased the expression of cleaved-PARP, P53, and Bax. Method: H460 cells were treated with B82 at the indicated concentrations for 24 h, and then were harvested for protein extraction. The protein levels of cleaved-PARP, P53, and Bax were detected by western blot analysis using antibodies from Santa Cruz Biotechnology (*Santa Cruz, CA*). Representative western blots were shown from three independent experiments. Click here for file
