# Supplementary material for: Evaluation of a curcumin analog as an anti-cancer agent inducing ER stress-mediated apoptosis in non-small cell lung cancer cells
Source: BMC Cancer. 2013 Oct 24;13:494. doi: 10.1186/1471-2407-13-494 (PMC4015692; doi:10.1186/1471-2407-13-494)
Supplement: Additional file 1: Table S1 — Chemical information and sources of the tested 113 compounds. Figure S1. B82 treatment increased the expression of cleaved-PARP, P53, and Bax. Method: H460 cells were treated with B82 at the indicated concentrations for 24 h, and then were harvested for protein extraction. The protein levels of cleaved-PARP, P53, and Bax were detected by western blot analysis using antibodies from Santa Cruz Biotechnology (Santa Cruz, CA). Representative western blots were shown from three independent experiments. [file 1471-2407-13-494-S1.doc]

**Supporting Information**

**Evaluation of a curcumin analog as an anti-cancer agent inducing ER stress-mediated apoptosis in non-small cell lung cancer cells**

Yi Wang^1,#^, Zhiguo Liu^1,#^, Xiuhua Zhang^2^, Luqing Ren^1^, Yi Huang^1^, Yuepiao Cai^1^, Xueqian Shen^1^, Xiaokun Li^1, *^, Guang Liang^1, *^

^1^ Chemical Biology Research Center, School of Pharmacy, Wenzhou Medical University, University Town, Wenzhou, Zhejiang, China

^2^ Department of Pharmacy, the First Affiliated Hospital of Wenzhou Medical University, Wenzhou, Zhejiang, China

**Table S1**. Chemical information and sources of the tested 113 compounds

| **Comp.** | **Structure** | **Ref.** |  | **Comp.** | **Structure** | **Ref.** |
| --- | --- | --- | --- | --- | --- | --- |
| A67 |  |  |  | 1 |  | [3] |
| A68 |  |  |  | 2 |  |  |
| A69 |  |  |  | 3 |  |  |
| A70 |  |  |  | 4 |  |  |
| A72 |  |  |  | 5 |  |  |
| A73 |  |  |  | 6 |  |  |
| A74 |  |  |  | 7 |  |  |
| A75 |  |  |  | 8 |  |  |
| B67 |  |  |  | 9 |  |  |
| B68 |  |  |  | 10 |  |  |
| B69 |  |  |  | 11 |  |  |
| B72 |  |  |  | 12 |  |  |
| B73 |  |  |  | 13 |  |  |
| B74 |  |  |  | 14 |  |  |
| B75 |  |  |  | 15 |  |  |
| C67 |  |  |  | 16 |  |  |
| C68 |  |  |  | 17 |  |  |
| C69 |  |  |  | 18 |  |  |
| C70 |  |  |  | 19 |  |  |
| C72 |  |  |  | 20 |  |  |
| C73 |  |  |  | 21 |  |  |
| C74 |  |  |  | 22 |  |  |
| C75 |  |  |  | 23 |  |  |
| A76 |  |  |  | 24 |  |  |
| A77 |  |  |  | 25 |  |  |
| A78 |  |  |  | 26 |  |  |
| A80 |  |  |  | 27 |  |  |
| A81 |  |  |  | 28 |  |  |
| A82 |  |  |  | 29 |  |  |
| A84 |  |  |  | 30 |  |  |
| A86 |  |  |  | 31 |  |  |
| A88 |  |  |  | 32 |  |  |
| NA1 |  |  |  | 33 |  |  |
| NA2 |  |  |  | 34 |  |  |
| NA3 |  |  |  | 35 |  |  |
| NA4 |  |  |  | 36 |  |  |
| B76 |  |  |  | 37 |  |  |
| B78 |  |  |  | 38 |  |  |
| B82 |  |  |  | 39 |  |  |
| B84 |  |  |  | 40 |  |  |
| B86 |  |  |  | 41 |  |  |
| NB1 |  |  |  | 42 |  |  |
| NB2 |  |  |  | 43 |  |  |
| NB3 |  |  |  | 44 |  |  |
| NB4 |  |  |  | 45 |  |  |
| C77 |  |  |  | 46 |  |  |
| C78 |  |  |  | 47 |  |  |
| C80 |  |  |  | 48 |  |  |
| C81 |  |  |  | 49 |  |  |
| C82 |  |  |  | 50 |  |  |
| C84 |  |  |  | 51 |  |  |
| C86 |  |  |  | 52 |  |  |
| C87 |  |  |  | 53 |  |  |
| NC1 |  |  |  | 54 |  |  |
| NC2 |  |  |  | C02 |  | [4] |
| NC3 |  |  |  |  |  |  |
| NC4 |  |  |  |  |  |  |


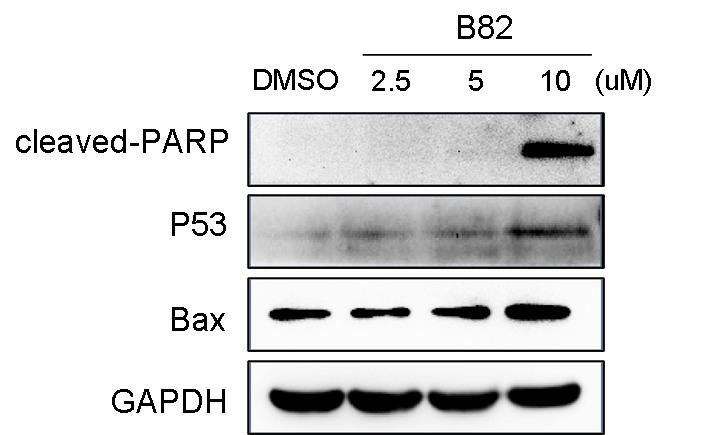


Figure S1. B82 treatment increased the expression of cleaved-PARP, P53, and Bax. Method: H460 cells were treated with B82 at the indicated concentrations for 24 h, and then were harvested for protein extraction. The protein levels of cleaved-PARP, P53, and Bax were detected by western blot analysis using antibodies from Santa Cruz Biotechnology (*Santa Cruz, CA*). Representative western blots were shown from three independent experiments.

**References:**

1. Zhao C, Yang J, Wang Y, Liang D, Yang X, Li X, Wu J, Wu X, Yang S, Liang G: **Synthesis of mono-carbonyl analogues of curcumin and their effects on inhibition of cytokine release in LPS-stimulated RAW 264.7 macrophages**. *BioorgMedChem* 2010.

2. Zhao C, Cai Y, He X, Li J, Zhang L, Wu J, Zhao Y, Yang S, Li X, Li W *et al*: **Synthesis and anti-inflammatory evaluation of novel mono-carbonyl analogues of curcumin in LPS-stimulated RAW 264.7 macrophages**. *Eur J Med Chem* 2010, **45**(12):5773-5780.

3. Wu J, Li J, Cai Y, Pan Y, Ye F, Zhang Y, Zhao Y, Yang S, Li X, Liang G: **Evaluation and discovery of novel synthetic chalcone derivatives as anti-inflammatory agents**. *J Med Chem* 2011, **54**(23):8110-8123.

4. Liang G, Li X, Chen L, Yang S, Wu X, Studer E, Gurley E, Hylemon PB, Ye F, Li Y *et al*: **Synthesis and anti-inflammatory activities of mono-carbonyl analogues of curcumin**. *Bioorg Med Chem Lett* 2008, **18**(4):1525-1529.
